# Metabolomics Analysis of the Larval Head of the Silkworm, *Bombyx mori*

**DOI:** 10.3390/ijms17091460

**Published:** 2016-09-20

**Authors:** Yi Li, Xin Wang, Quanmei Chen, Yong Hou, Qingyou Xia, Ping Zhao

**Affiliations:** 1 State Key Laboratory of Silkworm Genome Biology, Southwest University, Chongqing 400716, China; yili89716@gmail.com (Y.L.); wangxin1-4@163.com (X.W.); yhou@swu.edu.cn (Y.H.); xiaqy@swu.edu.cn (Q.X.); 2 Department of Biochemistry & Molecular Biology, Chongqing Medical University, Chongqing 400016, China; eidoloncqm@126.com

**Keywords:** metabolites, pathway analysis, GC-MS, LC-MS/MS, head, silkworm

## Abstract

The head, which performs many biological functions, is the most complicated structure of an insect. Development, locomotor behavior, food intake, environmental sensing, and signal transduction are all controlled by the insect’s head. As a well-studied insect in Lepidoptera, the silkworm head has an additional function of spinning silk fibers. To understand which molecules are involved in these physiological activities, we performed a metabolomics analysis of silkworm heads. By integrating GC-MS and LC-MS/MS, 90 metabolites were identified in the larval heads of silkworms. These were classified into 13 categories, including amino acids, sugars, organic acids, nucleotides, alcohols, and fatty acids. Informatics analysis revealed that these metabolites are involved in cellular processes, environmental information processing, genetic information processing, human diseases, metabolism, organismal systems, and other pathways. The identified metabolites and pathways are involved in biological processes such as signal transduction, carbohydrate metabolism, endocrine activities, and sensory activities; reflecting the functions of various organs in silkworm heads. Thus, our findings provide references which elucidate the potential functions of the silkworm head and will be of great value for the metabolomics research of silkworms and other insects.

## 1. Introduction

The silkworm, *Bombyx mori*, is an important economic insect. It is also a well-studied insect in the Lepidoptera. The silkworm body consists of a series of somites, which are grouped into three segments called the cephalosome, thorax, and abdomen [1]. The cephalosome, often termed the head, is a complex structure containing the most complicated endocrine system in insects; comprising of the brain, suboesophageal ganglion (SG), and corpora allatum, which controls multiple biological behaviors. Other organs, such as the antennae, ocellasae, mouthparts, and spinnerets are also attached to the silkworm head. These diverse organs contribute to making the head a multi-functional body part.

Microarray analyses have identified 6635 active genes in silkworm heads, including 78 head-specific genes that encode proteins such as opsin, odorant binding protein, chemosensory protein, odorant-degrading esterase, diapause protein 1, and ecdysteroid-regulated protein [2]. Furthermore, 539 proteins in silkworm heads have been characterized by shotgun technology using liquid chromatography, in combination with tandem mass spectrometry (LC-MS/MS). These include chemosensory proteins, odorant-binding proteins, diapause-related proteins, and cuticle proteins [3]. Kyoto Encyclopedia of Genes and Genomes (KEGG) analysis showed that many of these proteins are involved in metabolic pathways and human neurodegenerative diseases [3].

The metabolic profiles of living organisms reflect features of their life activities. By measuring and mathematically modelling the metabolic changes in biological fluids or tissues, metabolomics offers fresh insight into the effects of many stimuli or stresses [4]. Nuclear magnetic resonance (NMR), gas chromatography with mass spectrometry (GC-MS), and LC-MS/MS are the most widely used methods for metabolomics research [5,6,7].

In recent years, metabolomics analysis has been used in silkworm studies, including those examining hemolymph [8,9] and larval brains [10], by using NMR or GC-MS. Nutrient balance [8], individual development [9], and locomotor behavior [10] of the silkworm have been clarified by metabolomics analysis. However, a study of the metabolic profile of a complex structure such as the silkworm head has, to the best of our knowledge, never been reported.

Due to different technical characteristics, a specific compound can often only be identified by either GC-MS or LC-MS/MS. Since it is typically not sufficient to use single techniques to identify the metabolic profile in organism tissues, we combined GC-MS and LC-MS/MS in order to analyze the larval heads of silkworms to determine the complete metabolic profile. The combination of our metabolomics profiling with the reported transcriptome [2] and proteome [3] data, provides a systems biological approach to the study of silkworm heads, which may offer a comprehensive reference to study the function of the silkworm head. In summary, our data will provide a better understanding of insect head metabolomics and provide a preferable foundation for further studies of silkworm metabolomics and physiological functions of the insect’s head.

## 2. Results and Discussion

### 2.1. Metabolites Identified by GC-MS and LC-MS/MS

**Figure 1:**
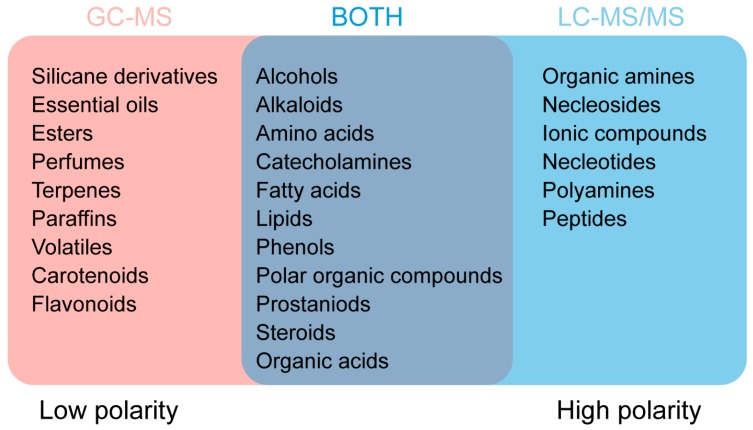
Compounds that can be identified using GC-MS and LC-MS/MS.

A total of 90 metabolites were identified by GC-MS and LC-MS/MS in the larval heads of silkworms (Table 1). The total ion chromatogram (TIC) from GC-MS and LC-MS/MS was shown in Figures S1 and S2, respectively. Sixty metabolites were identified by LC-ESI-MS/MS and 30 of them were identified by GC-EI-MS. The ESI is a soft ionization technique that is mainly used for LC-MS/MS. It is suitable for analyzing compounds that have large molecular weight, low stability or strong polarity. Electron ionization (EI) is the most widely used method for ionization. Generally, it is used for analyzing volatile samples [11]. On the basis of our data, and those of metabolomics studies using mass spectrometry [8,10,12,13], an overview of the approximate range of applications of GC-MS (EI+) and LC-MS/MS (ESI+/−) is given in Figure 1. GC-MS can be used for identifying metabolites with low polarity such as silicane derivatives, esters, perfumes, and volatiles, whereas LC-MS/MS is suitable for identifying some high polarity metabolites such as organic amines, nucleosides, nucleotides, and ionic compounds. For compounds with diverse characteristics, such as alcohols, amino acids, fatty acids, and organic acids; GC-MS and LC-MS/MS can both be used for identification (Figure 1).

**Figure 2:**
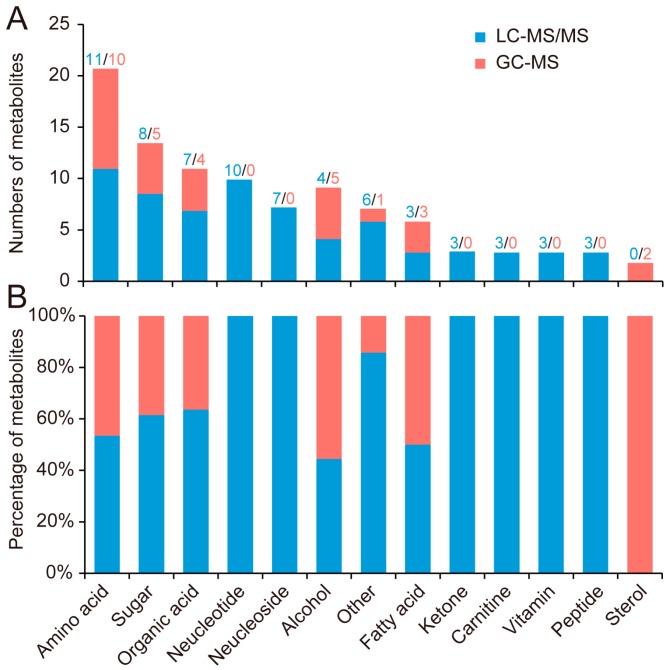
Classification of the identified metabolites. (**A**) The numbers of metabolites identified by GC-MS or LC-MS/MS; (**B**) The percentage of metabolites identified by GC-MS and LC-MS/MS.

On the basis of certain features of the 90 identified metabolites, they were classified into 13 categories: amino acid, sugar, organic acid, nucleotide, nucleoside, alcohol, fatty acid, ketone, carnitine, vitamin, peptide, sterol, and other metabolites (Figure 2).

Previous metabolomics studies on the silkworm hemolymph [8] and brain [10] both showed that amino acids are the most common class of metabolites. In our data, 21 amino acids were identified and accounted for almost 25% of the identified metabolites. Among them, 11 amino acids were identified by LC-MS/MS, whereas 10 amino acids were identified by GC-MS (Table S1). Similarly, sugars, organic acids, alcohols, and fatty acids were identified by both GC-MS and LC-MS/MS.

Nucleosides, nucleotides, ketones, carnitines, vitamins, and peptides were only identified by LC-MS/MS. Because of their high polarity and the difficulty of gasification, nucleosides and nucleotides can be identified by LC-MS/MS. We also identified two peptides—glutathione and glutathione disulfide—using LC-MS/MS (Table 1). Consisting of glutamate, cysteine and glycine; glutathione (GSH) is an important antioxidant in living organisms [14]. Glutathione disulfide (GSSG) is a disulfide derived from two GSH molecules [15]. In our study, GSH was identified in both ESI+ and ESI- mode, whereas GSSG was only identified in ESI+ mode (Table 1).

Sterols, including cholesterol and β-sitosterol, were only identified by GC-MS. In the silkworm, cholesterol is the substrate of ecdysone [16]. It cannot, however, be synthesized by the silkworms themselves but is extracted from their mulberry leaf diet [17]. β-sitosterol in silkworms is also extracted from mulberry leaves and can be converted into cholesterol in vivo [17].

**Figure 3:**
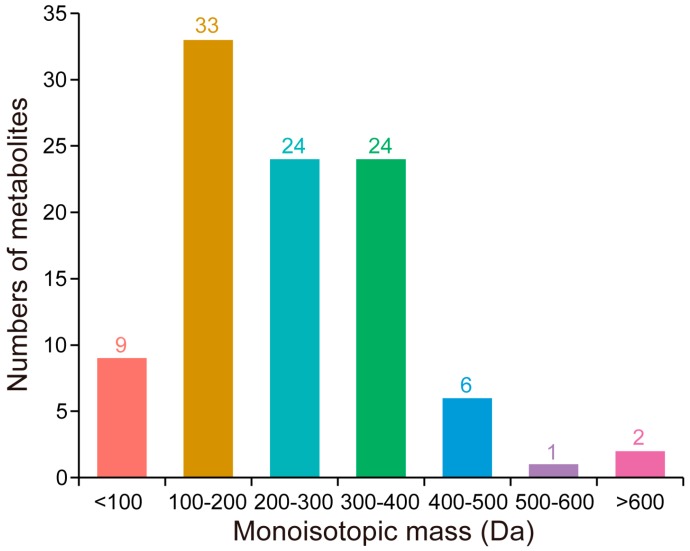
Monoisotopic mass distribution of the identified metabolites.

By searching against the database, the monoisotopic mass of the identified metabolites ranged from 46 to 650 Da (Table S1). On the basis of their monoisotopic mass, the metabolites were divided into seven groups (Figure 3). Most of these (81.82%) had a monoisotopic mass ranging from 100 to 400 Da.

### 2.2. Pathway Analysis

**Figure 4:**
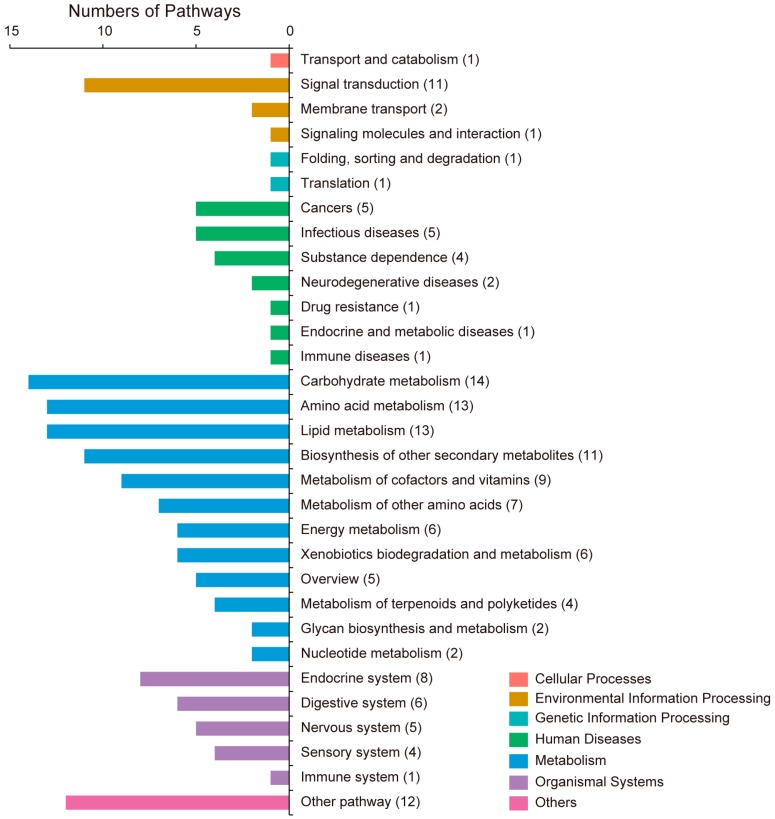
Categories of pathways in which the identified metabolites are involved.

Pathways are required for the maintenance of homeostasis within organisms. The amount of metabolites in a pathway is regulated by the requirements of cells and the availability of the substrates. The end product of a pathway may be used immediately, participate in another pathway or be stored for later use [18,19]. The metabolites identified in this study were submitted to the KEGG pathway database to obtain the pathway ID; 164 of these pathways were identified (Table S2). The pathways were classified into seven categories, namely; cellular processes, environmental information processing, genetic information processing, human diseases, metabolism, organismal systems, and others (Figure 4).

Among the seven categories, the most abundant was “metabolism”. Metabolism is a set of biochemical transformations in the living cells. These biochemical transformations allow organisms to grow, reproduce, maintain their physiological structures, and respond to the changing environment. In our data, the top three pathway categories in “metabolism” were carbohydrate metabolism, amino acid metabolism and lipid metabolism (Figure 4). One of the important functions of carbohydrate and lipid metabolism is to provide energy for living cells. Our results revealed that many of the pathways were classified into carbohydrate metabolism (14 pathways) or lipid metabolism (13 pathways), indicating that the silkworm head requires large amounts of energy. A further 13 pathways were classified into amino acid metabolism, which suggests that many proteins are degraded or synthesized in silkworm heads. Moreover, many pathways were classified into the biosynthesis of other secondary metabolites; the metabolism of cofactors and vitamins, the metabolism of other amino acids and the energy metabolism.

Sensing the environment is a vital function of the silkworm head. There are four types of sensory organs in silkworm heads, namely; the tactile, olfactory, gustatory, and ocular organs. These sensory organs help silkworms to sense environmental stimuli and make responses [1]. Tactile organs, also called mechanical sense organs, are primarily responsible for sensing the stimulation from the contact with other objects. They can also sense temperature, airflow, and air pressure. Antennae and maxillary palps are the primary tactile organs in the silkworm head [20]. Olfactory organs are used for detecting gaseous chemicals; therefore, they are also referred to as chemical receptors. The foramen olfactorium is the most important olfactory organ of the silkworm, and almost 70% of the foramen olfactorium is located in the silkworm head [21]. Gustatory organs are responsible for receiving liquid chemical stimuli, and they are mainly distributed on the silkworm mouthparts [22]. Ocular organs, which include ocellasae (larval stage) and compound eyes (adult stage), are involved in the reception of photo stimulation [23]. Microarray analysis of various silkworm tissues showed that there are 78 head-specific gene products, such as opsin, which regulates retinal signal transduction, odorant-binding protein, and chemosensory protein; odorant-degrading esterase are responsible for sensory signaling [2]. By using metabolomics techniques, we identified four pathways in the sensory system, namely; olfactory transduction, taste transduction, photo transduction, and inflammatory mediator regulation of transient receptor potential (TRP) channels (Table S2). These results are consistent with the function of the sensory organs in silkworm heads.

Parts of the central nervous system, such as the brain and SG, are also located in the silkworm head. The brain is the control center of silkworms [10,24], whereas the SG is responsible for secreting diapause hormones [25]. Genes encoding diapause protein 1 and ecdysteroid-regulated protein were identified in silkworm heads, which may be related to steroid hormone regulation [2]. In this study, 13 pathways were classified into the endocrine system and nervous system (Figure 4), whereas 11 pathways were classified into signal transduction in environmental information processing (Figure 4), which reflect the functions of the brain and the SG.

A further 19 of the identified pathways are involved in human diseases (Figure 4). A previous study of the proteomics of the silkworm head showed that it contains many proteins that are involved in human disease pathways, and accordingly, the silkworm could potentially be used as another model for human disease research [3]. Our results further confirmed this supposition.

### 2.3. Possible Functions of the Identified Metabolites

As the central controlling organ of the silkworm, the brain is located in the middle of the head. In our previous study, 37 metabolites were identified in the silkworm larval brain using GC-MS [10]. Among these 37 metabolites, 25 were also identified in the silkworm larval head in the present study (Table 1). Dopamine is one of the most important neurotransmitters in the brain [10,26,27,28], it can control many behaviors such as locomotor, reproduction, aggression, diapause, and hormone regulation. Furthermore, it is difficult to identify using GC-MS or normal reverse-phase (C18 column) LC-MS/MS due to its high polarity [29]. However, using LC-MS/MS, we identified l-dopa—the precursor of dopamine [30] (Table 1)—which can convert into dopamine to participate in those biological functions. Furthermore, we identified certain small molecules such as l-tryptophan (the precursor of 5-hydroxytryptamine), glycine, serine, and CDP-choline. Many authors have concluded that these molecules may participate in signal transduction as neurotransmitters [31,32,33,34]. Thus, our data suggests that silkworm heads are actively involved in signal transduction. Further analysis of these molecules would be helpful in revealing the details of signal transduction in silkworms.

The silkworm is also an important economic insect due to its production of silk fibers. Synthesized by the silk gland, the silk fiber is spun out of spinnerets, which are located in the head [35]. A transcriptome study of silkworm spinnerets showed that many genes are related to metabolic pathways [36]. To further analyze the function of spinnerets in the silk spinning process, KEGG enrichment was performed between the spinning stage and feeding stage. The results showed that there are seven enriched metabolic pathways: carbon metabolism, citrate cycle, biosynthesis of amino acids, pyruvate metabolism, metabolism pathways, 2-oxocarboxylic acid metabolism, and amino sugar and nucleotide sugar metabolism. All of these enriched pathways were also identified in our metabolome data (Table S2). These findings indicate that the metabolites involved in these pathways might participate in the spinning process. 

A previous study has revealed that 272 proteins with EC (Enzyme Commission) numbers are involved in 73 KEGG pathways in the silkworm head, and more than half of the identified pathways are related to metabolic processes [3]. Among these, amino acid metabolism and carbohydrate metabolism are the most active pathways. Insect heads are filled with muscles and organs such as the brain, SG, corpora allatum, and spinnerets [37], which need large amounts of energy to maintain their physiological functions [38,39]. Furthermore, it has been proposed that silk fiber formation in the spinnerets is an energy-consuming process [36]. Therefore, metabolic processes in silkworm heads should be active. We found large amounts of metabolites involved in the carbohydrate, lipid, and energy metabolism (Figure 4). These metabolites may provide a rich energy supply for the signal transduction, hormonal secretion, muscle concentration, and silk fiber formation in silkworm heads.

## 3. Materials and Methods

### 3.1. Insect Rearing and Sample Preparation

Silkworms, strain *Dazao* (P50), were reared on fresh mulberry leaves at 26 ± 1 °C and 70% ± 10% relative humidity. Larval heads were dissected on the third day of the fifth instar and immediately stored at −80 °C. Each group (with 3 biological replicates) containing 30 heads was homogenized in a mortar with liquid nitrogen. Powdered samples (100 mg) from each group were suspended with a 500 μL methanol-water (*v*/*v*, 4:1) mixture and sonicated for 20 min, followed by incubation on ice for 40 min. Thereafter, the samples were centrifuged at 15,000× *g* for 30 min. The supernatant was transferred to a centrifuge tube and lyophilized for 5 h in a vacuum concentrator [10]. The lyophilized samples were then re-dissolved in 65 μL pyridine (containing 15 mg/mL methoxylamine hydrochloride; Sigma-Aldrich, St. Louis, MO, USA) for GC-MS analysis or in a 80 μL methanol-water (*v*/*v*, 4:1) mixture for LC-MS/MS analysis.

### 3.2. GC-MS Analysis

Samples dissolved in pyridine were incubated at 37 °C for 1.5 h and silanized for 1 h at 37 °C with the addition of 80 μL *N*-methyl-*N*-(trimethylsilyl) trifluoroacetamide (MSTFA). The reaction was then stopped by adding 10 μL hexane into the samples. The derivative sample was auto-injected into an Agilent 7890B/5977A GC-MS system (Agilent, San Jose, CA, USA), which was equipped with a DB-5MS column (length 30 m, i.d. 0.25 mm; Agilent), and the injection temperature was held at 300 °C. The oven temperature program was set as follows: initially kept at 60 °C for 3 min, 5 °C·min^−1^ to 170 °C, 4 °C·min^−1^ to 234 °C, 5 °C·min^−1^ to 270 °C, then heated to 300 °C at 10 °C·min^−1^, and held for 5 min [10]. The voltage of the detector was set to 0.93 kV, and the EI ionization voltage of the metabolites was 70 eV. Mass spectra were recorded from *m*/*z* 50 to *m*/*z* 450. Peaks were identified through spectral matching against standards from the National Institute of Standards and Technology (NIST) standard database, and a relative score greater than 700 was considered to be a good match.

### 3.3. LC-MS/MS Analysis

A 10-μL aliquot of each sample (prepared as described above) was auto-injected into a Thermo Scientific Ultimate 3000 UHPLC system (Thermo Fisher Scientific, Waltham, MA, USA). Chromatographic separation was achieved using an Agilent Zorbax C18 column (1.8 μm, 2.1 × 150 mm; Agilent). The flow phases used were (A) aqueous 0.1% (*v*/*v*) formic acid and (B) acetonitrile 0.1% (*v*/*v*) formic acid. The chromatographic conditions were as follows: 0–1 min, 2% B; 1–5 min, 2%–15% B; 5–13 min, 15%–70% B; 13–25 min, 70%–98% B; 25–30 min, 98% B; 30–30.2 min, 98%–2% B; 30.2–35 min, 2% B. High resolution accurate mass data was acquired both in positive and negative ion modes using a Thermo Scientific Q-Exactive Orbitrap mass spectrometer (Thermo Fisher Scientific) operated at a resolution of 70,000. The voltage of the electrospray source was set to 3.5 kV in the positive mode (ESI+) and 2.8 kV in the negative mode (ESI−). Mass spectra were recorded from *m*/*z* 100 to *m*/*z* 1000. The raw data was analyzed using the Component Extraction algorithm in SIEVE 2.0 software (Thermo Fisher Scientific) to detect the metabolites. The intensity threshold was set to 3,000,000, and four databases were chosen to identify the metabolites: the BioCyc Pathway, KEGG, Lipid Maps Databases, and NIST. The MZ tolerance was set to 5 ppm for the database search.

### 3.4. Statistics and Informatics Analysis

The identified metabolites were first submitted to the KEGG pathway database (available online: http://www.kegg.jp/kegg/pathway.html) to obtain the KEGG ID and the pathway ID. Those small molecules that could not be identified from the KEGG pathway database were classified as unidentified metabolites and were not used for further analysis. The monoisotopic mass of the identified metabolites was obtained from the ChemSpider website (available online: http://www.chemspider.com/).

## 4. Conclusions

In this study, we integrated GC-MS and LC-MS/MS to analyze the silkworm larval head, which enabled us to further understand the metabolic profile of insect larval heads. In our data, 90 metabolites and 164 related pathways were identified. Combining previous studies with our metabolome data, the multi-functions of the silkworm head become clearer. The identified metabolites and pathways involved in biological processes such as signal transduction, carbohydrate metabolism, endocrine system, and sensory system may reflect the functions of various organs in silkworm heads. This study represents the first detailed investigation of silkworm heads’ metabolomics using GC-MS and LC-MS/MS. As a consequence, our results can provide novel insights and references into silkworm metabolomics and a functional study of the insect’s head.

## Figures and Tables

**Table 1:** The metabolites identified by GC-MS and LC-MS/MS.

Metabolite Name	KEGG ID	Metabolite Name	KEGG ID
†d-Mannitol 1-phosphate *	C00644	†Uridine *	C00299
‡d-Sorbitol	C00794	†Uridine monophosphate	C00105
‡Ethanol	C00469	†Xanthosine 5′-phosphate	C00655
‡Glycerol	C00116	‡(9*Z*,12*Z*,15*Z*)-Octadecatrienoic acid	C06427
‡Mannitol	C00392	‡(*S*)-Lactate	C00186
‡myo-Inositol	C00137	†13(*S*)-HPOT	C04785
†sn-Glycero-3-phosphocholine	C00670	†3-Deoxy-d-manno-octulosonate 8-phosphate	C04478
†sn-Glycerol 3-phosphate	C00093	†Allantoate	C00499
†3-Hydroxy-l-kynurenine	C03227	‡Butanoic acid	C00246
‡β-Alanine	C00099	†Citrate	C00158
†D-Phenylalanine	C02265	‡Glutarate	C00489
‡Glycine	C00037	‡Hexadecanoic acid	C00249
‡l-Alanine	C00041	†Nogalonate	C12416
†l-Arginine	C00062	†Phenylpyruvate	C00166
‡l-Asparagine	C00152	‡Propanoate	C00163
†l-Histidine	C00135	†Stearidonic acid	C16300
‡l-Isoleucine	C00407	‡Succinate	C00042
‡l-Leucine	C00123	†Tetracosanoic acid	C08320
†l-Methionine	C00073	†Urate	C00366
‡l-Proline	C00148	†Urate-3-ribonucleoside	C05513
‡Serine	C00065	†2-Phytyl-1,4-naphthoquinone	C13309
‡l-Threonine	C00188	†CDP-choline	C00307
†l-Tryptophan	C00078	†Dihydroneopterin phosphate	C05925
‡l-Valine	C00183	†l-Dopa	C00355
†*N*-Acetyl-l-glutamate *	C00624	†OPC-8:0	C04780
†Protein N6-methyl-l-lysine	C05544	†Retinyl palmitate	C02588
†*S*-Adenosyl-l-homocysteine	C00021	‡Urea	C00086
†*S*-Adenosyl-l-methionine	C00019	†Glutathione *	C00051
†Carnitine	C00487	†Glutathione disulfide	C00127
†l-Palmitoylcarnitine	C02990	‡Cholesterol	C00187
†*O*-Propanoylcarnitine	C03017	‡β-Sitosterol	C01753
†Androsterone	C00280	†Acetyl-maltose	C02130
†Solavetivone	C09737	‡α,α-Trehalose	C01083
†Umbelliferone	C09315	†α-d-Glucose 6-phosphate	C00668
†5′-Methylthioadenosine	C00170	‡d-Allose	C01487
†7-Aminomethyl-7-carbaguanine	C16675	†d-Arabinose 5-phosphate	C01112
†Adenosine diphosphate *	C00008	†d-Glucosamine 6-phosphate	C00352
†Adenosine monophosphate *	C00020	‡d-Lyxose	C00476
†Cytidine	C00475	‡d-Talose	C06467
†Guanine	C00242	†*N*-Acetyl-d-glucosamine 6-phosphate	C00357
†Guanosine *	C00387	†Sedoheptulose 7-phosphate	C05382
†Guanosine monophosphate	C00144	†Trehalose 6-phosphate	C00689
†Inosine	C00294	‡Turanose	C19636
†Triiodothyronine	C02465	†Xylobiose	C01630
†Thiamine	C00378	†Riboflavin *	C00255

† Metabolites identified by LC-MS/MS; ‡ Metabolites identified by GC-MS; * Metabolites identified by LC-MS/MS using both ESI+ and ESI− modes.

## Supplementary Materials

Supplementary materials can be found at www.mdpi.com/1422-0067/17/9/1460/s1.
