# Supplementary material for: Metabolomics Analysis of the Larval Head of the Silkworm, Bombyx mori
Source: Int J Mol Sci. 2016 Sep 20;17(9):1460. doi: 10.3390/ijms17091460 (PMC5037739; doi:10.3390/ijms17091460)
Supplement: Supplementary file 1 [file ijms-17-01460-s001.pdf]

# Supplementary Materials: Metabolomics Analysis of the Larval Head of the Silkworm, *Bombyx mori*

**Yi Li, Xin Wang, Quanmei Chen, Yong Hou, Qingyou Xia and Ping Zhao**

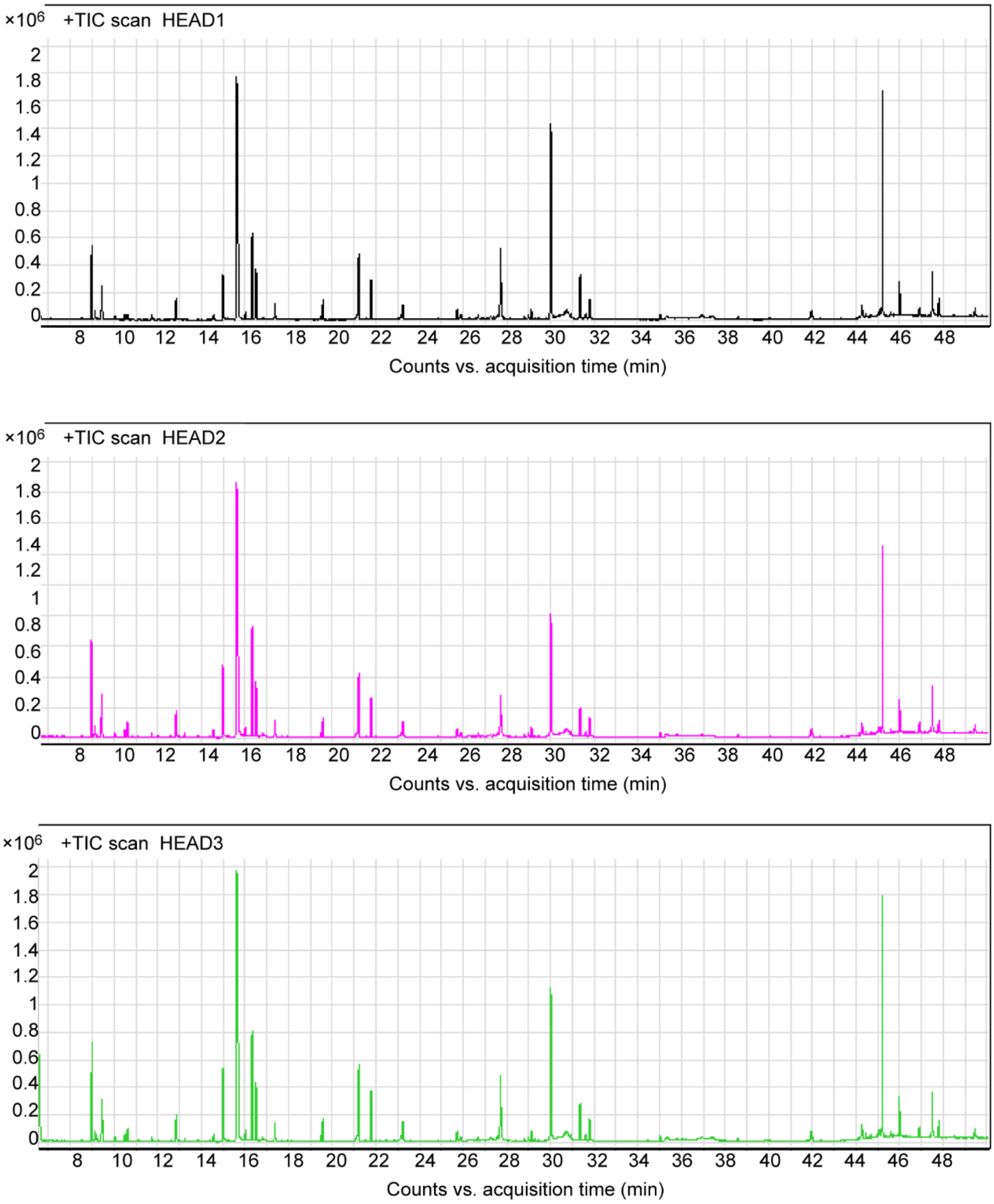


**Figure S1.** The total ion chromatogram from GC-MS.

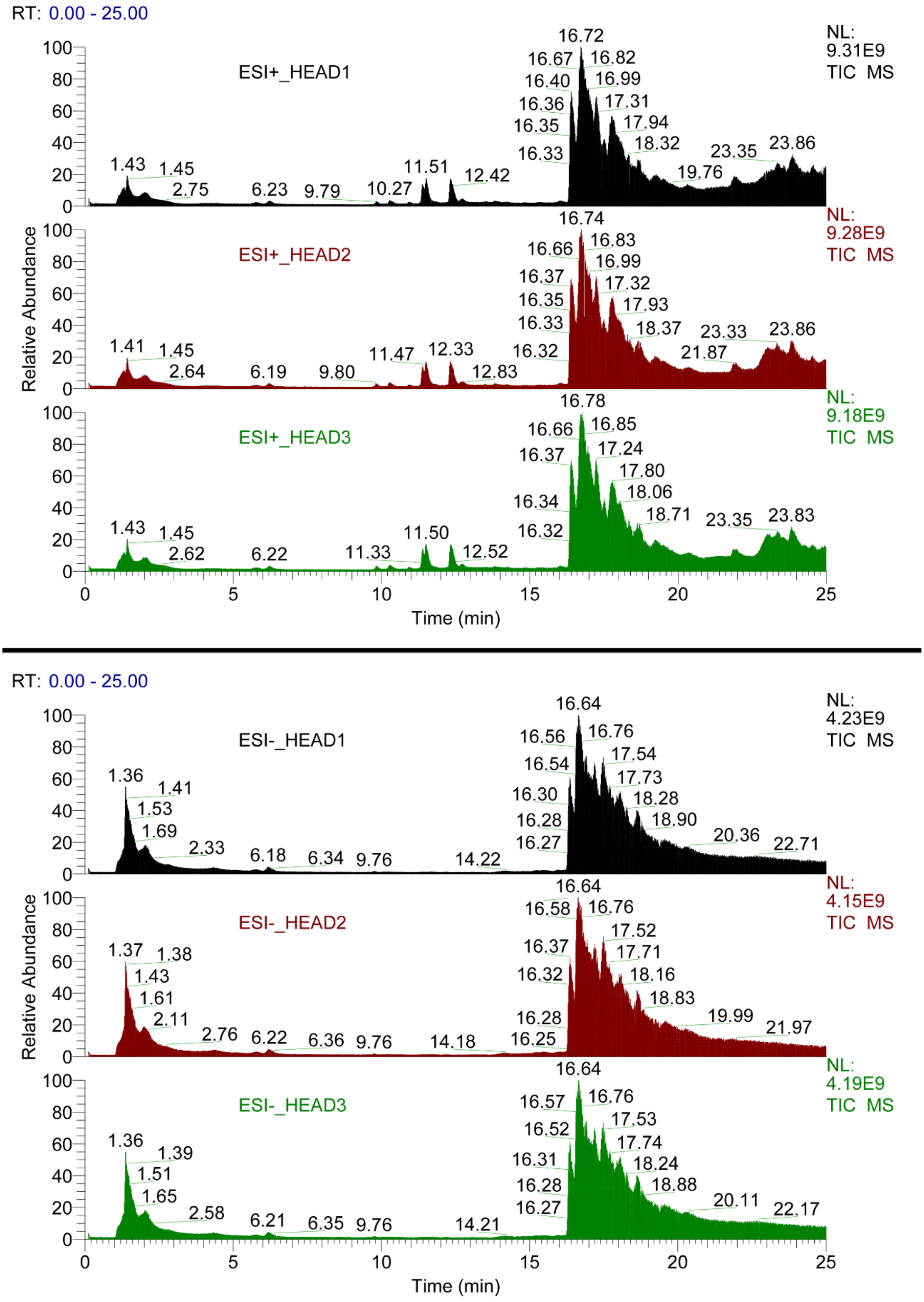


**Figure S2.** The total ion chromatogram from LC-MS/MS.

**Table S1.** Detailed information of the metabolites identified by GC-MS and LC-MS/MS.

| Classification | Metabolite Name | KEGG ID | Mass-to-charge Ratio [*m*/*z*] | Retention Time [min] | Ion Source | Monoisotopic Mass [Da] | DeltaPPM | Relative Score |
|---|---|---|---|---|---|---|---|---|
| Alcohol | D-Mannitol 1-phosphate | C00644 | 261.042831 | 1.50 | ESI− | 260.030823 | 0.166279 | - |
| Alcohol | sn-Glycerol 3-phosphate | C00093 | 173.021149 | 1.72 | ESI+ | 172.013672 | 1.163172 | - |
| Alcohol | sn-Glycero-3-phosphocholine | C00670 | 258.109772 | 1.41 | ESI+ | 257.102814 | 1.240250 | - |
| Alcohol | D-Mannitol 1-phosphate | C00644 | 263.052673 | 1.63 | ESI+ | 260.030823 | 0.063881 | - |
| Alcohol | Glycerol | C00116 | 205.1 | 15.68 | EI+ | 92.04734 | - | 803 |
| Alcohol | Mannitol | C00392 | 319.1 | 31.62 | EI+ | 182.079041 | - | 727 |
| Alcohol | myo-Inositol | C00137 | 319.1 | 31.80 | EI+ | 180.063385 | - | 713 |
| Alcohol | D-Sorbitol | C00794 | 319.1 | 31.79 | EI+ | 182.079041 | - | 846 |
| Alcohol | Ethanol | C00469 | 267.1 | 27.41 | EI+ | 46.041866 | - | 711 |
| Amino acid | *N*-Acetyl-L-glutamate | C00624 | 188.052948 | 2.24 | ESI− | 189.063721 | 2.109705 | - |
| Amino acid | L-Methionine | C00073 | 150.058334 | 1.50 | ESI+ | 149.051056 | 0.011745 | - |
| Amino acid | L-Histidine | C00135 | 156.077011 | 1.13 | ESI+ | 155.069473 | 1.686392 | - |
| Amino acid | Protein N6-methyl-L-lysine | C05544 | 161.128799 | 1.13 | ESI+ | 160.121185 | 2.109888 | - |
| Amino acid | D-Phenylalanine | C02265 | 166.086395 | 4.03 | ESI+ | 165.078979 | 0.846041 | - |
| Amino acid | L-Arginine | C00062 | 175.119217 | 1.13 | ESI+ | 174.111679 | 1.500867 | - |
| Amino acid | *N*-Acetyl-L-glutamate | C00624 | 190.071259 | 2.19 | ESI+ | 189.063721 | 1.380194 | - |
| Amino acid | L-Tryptophan | C00078 | 205.097229 | 5.72 | ESI+ | 204.089874 | 0.384163 | - |
| Amino acid | 3-Hydroxy-L-kynurenine | C03227 | 225.086914 | 2.10 | ESI+ | 224.079712 | 0.332638 | - |
| Amino acid | L-Valine | C00183 | 72.1 | 10.05 | EI+ | 117.078979 | - | 865 |
| Amino acid | L-Alanine | C00041 | 116.1 | 10.63 | EI+ | 89.047676 | - | 890 |
| Amino acid | Glycine | C00037 | 102.0 | 11.11 | EI+ | 75.032028 | - | 787 |
| Amino acid | L-Leucine | C00123 | 86.1 | 12.03 | EI+ | 131.094635 | - | 717 |
| Amino acid | L-Isoleucine | C00407 | 86.1 | 12.60 | EI+ | 131.094635 | - | 703 |
| Amino acid | L-Threonine | C00188 | 73.0 | 16.03 | EI+ | 119.058243 | - | 775 |

| | | | | | | | | |
|---|---|---|---|---|---|---|---|---|
| Amino acid | β-Alanine | C00099 | 248.1 | 19.48 | EI+ | 89.047676 | - | 811 |
| Amino acid | L-Proline | C00148 | 156.1 | 21.79 | EI+ | 115.063332 | - | 879 |
| Amino acid | L-Asparagine | C00152 | 73.0 | 29.13 | EI+ | 132.053497 | - | 702 |
| Amino acid | Serine | C00065 | 116.0 | 15.00 | EI+ | 105.042595 | - | 872 |
| Amino acid | *S*-Adenosyl- L-homocysteine | C00021 | 385.128784 | 2.15 | ESI+ | 384.121582 | 0.193742 | - |
| Amino acid | *S*-Adenosyl- L-methionine | C00019 | 399.144775 | 1.31 | ESI+ | 398.137238 | 0.655027 | - |
| Carnitine | Carnitine | C00487 | 162.112747 | 1.30 | ESI+ | 161.105194 | 1.716840 | - |
| Carnitine | *O*-Propanoylcarnitine | C03017 | 218.138794 | 2.57 | ESI+ | 217.131409 | 0.498985 | - |
| Carnitine | L-Palmitoylcarnitine | C02990 | 400.341827 | 19.48 | ESI+ | 399.334869 | 0.796844 | - |
| Fatty acid | Stearidonic acid | C16300 | 277.215851 | 17.49 | ESI+ | 276.208923 | 1.262705 | - |
| Fatty acid | Tetracosanoic acid | C08320 | 369.372437 | 23.73 | ESI+ | 368.365417 | 0.697885 | - |
| Fatty acid | Hexadecanoic acid | C00249 | 313.2 | 33.34 | EI+ | 256.240234 | - | 821 |
| Fatty acid | (9*Z*,12*Z*,15*Z*)-Octadecatrienoic acid | C06427 | 79.0 | 34.46 | EI+ | 278.224579 | - | 827 |
| Fatty acid | 13(*S*)-HPOT | C04785 | 309.223236 | 14.47 | ESI− | 310.214417 | 1.615659 | - |
| Fatty acid | Propanoate | C00163 | 174.0 | 9.11 | EI+ | 73.029503 | - | 833 |
| Ketone | Umbelliferone | C09315 | 163.039139 | 0.57 | ESI+ | 162.031693 | 1.044202 | - |
| Ketone | Solavetivone | C09737 | 219.174393 | 0.58 | ESI+ | 218.167068 | 0.220474 | - |
| Ketone | Androsterone | C00280 | 291.231537 | 20.21 | ESI+ | 290.224579 | 1.098236 | - |
| Nucleoside | Uridine | C00299 | 243.123047 | 1.42 | ESI− | 244.069534 | 1.256678 | - |
| Nucleoside | Guanosine | C00387 | 282.090393 | 2.75 | ESI− | 283.091675 | 1.555915 | - |
| Nucleoside | Guanine | C00242 | 152.056671 | 1.41 | ESI+ | 151.049408 | 0.089093 | - |
| Nucleoside | Cytidine | C00475 | 244.092407 | 1.41 | ESI+ | 243.085526 | 1.626481 | - |
| Nucleoside | Uridine | C00299 | 245.076813 | 2.17 | ESI+ | 244.069534 | 0.008785 | - |
| Nucleoside | Inosine | C00294 | 269.087860 | 1.42 | ESI+ | 268.08078 | 0.732961 | - |
| Nucleoside | Guanosine | C00387 | 284.098541 | 1.41 | ESI+ | 283.091675 | 1.449498 | - |
| Nucleotide | Uridine monophosphate | C00105 | 323.060150 | 2.33 | ESI− | 324.035858 | 2.490545 | - |
| Nucleotide | Adenosine monophosphate | C00020 | 346.082886 | 1.40 | ESI− | 347.06308 | 0.477833 | - |
| Nucleotide | Xanthosine 5'-phosphate | C00655 | 363.015259 | 1.37 | ESI− | 364.042023 | 2.059706 | - |

| | | | | | | | | |
|---|---|---|---|---|---|---|---|---|
| Nucleotide | Adenosine diphosphate | C00008 | 426.067932 | 2.44 | ESI- | 427.029419 | 2.389623 | - |
| Nucleotide | 7-Aminomethyl-7-carbaguanine | C16675 | 180.088135 | 5.40 | ESI+ | 179.080704 | 0.860872 | - |
| Nucleotide | 5′-Methylthioadenosine | C00170 | 298.096771 | 6.19 | ESI+ | 313.084473 | 0.250294 | - |
| Nucleotide | Adenosine monophosphate | C00020 | 348.069824 | 1.41 | ESI+ | 347.06308 | 1.533963 | - |
| Nucleotide | Guanosine monophosphate | C00144 | 364.064880 | 1.41 | ESI+ | 363.057983 | 1.044542 | - |
| Nucleotide | Adenosine diphosphate | C00008 | 428.036835 | 2.10 | ESI+ | 427.029419 | 0.325778 | - |
| Nucleotide | Triiodothyronine | C02465 | 651.796387 | 1.22 | ESI+ | 650.790039 | 1.427315 | - |
| Organic acid | Citrate | C00158 | 189.044235 | 1.36 | ESI− | 189.005173 | 0.168223 | - |
| Organic acid | Succinate | C00042 | 147.0 | 16.49 | EI+ | 116.012054 | - | 936 |
| Organic acid | Phenylpyruvate | C00166 | 165.054718 | 2.10 | ESI+ | 163.04007 | 0.569455 | - |
| Organic acid | Urate | C00366 | 169.035507 | 1.41 | ESI+ | 168.029434 | 0.627263 | - |
| Organic acid | Allantoate | C00499 | 177.061874 | 1.54 | ESI+ | 176.05455 | 0.271448 | - |
| Organic acid | Nogalonate | C12416 | 383.075989 | 1.32 | ESI+ | 380.054321 | 0.434033 | - |
| Organic acid | Butanoic acid | C00246 | 147.0 | 14.39 | EI+ | 88.052429 | - | 883 |
| Organic acid | (*S*)-Lactate | C00186 | 147.0 | 9.44 | EI+ | 89.024422 | - | 785 |
| Organic acid | Glutarate | C00489 | 73.1 | 23.24 | EI+ | 130.02771 | - | 812 |
| Organic acid | Urate-3-ribonucleoside | C05513 | 299.088409 | 4.44 | ESI− | 300.070587 | 1.265652 | - |
| Organic acid | 3-Deoxy-D-manno-octulosonate 8-phosphate | C04478 | 317.043884 | 1.52 | ESI− | 318.035187 | 0.617194 | - |
| Other | OPC-8:0 | C04780 | 293.233795 | 15.98 | ESI− | 294.219482 | 1.810336 | - |
| Other | Dihydroneopterin phosphate | C05925 | 334.032715 | 1.36 | ESI− | 335.06308 | 2.692369 | - |
| Other | L-Dopa | C00355 | 198.076126 | 1.41 | ESI+ | 197.068802 | 0.241026 | - |
| Other | 2-Phytyl-1,4-naphthoquinone | C13309 | 437.173874 | 16.36 | ESI+ | 436.334137 | 2.159811 | - |
| Other | CDP-choline | C00307 | 489.114807 | 1.36 | ESI+ | 488.10733 | 0.410829 | - |
| Other | Retinyl palmitate | C02588 | 524.236511 | 17.28 | ESI+ | 524.459351 | 1.681237 | - |
| Other | Urea | C00086 | 147.0 | 14.56 | EI+ | 60.032364 | - | 857 |
| Peptide | Glutathione | C00051 | 306.101379 | 1.45 | ESI− | 307.083801 | 0.839596 | - |
| Peptide | Glutathione | C00051 | 308.090790 | 1.41 | ESI+ | 307.083801 | 0.937221 | - |

| | | | | | | | | |
|---|---|---|---|---|---|---|---|---|
| Peptide | Glutathione disulfide | C00127 | 613.158936 | 1.41 | ESI+ | 612.151978 | 0.521199 | - |
| Sterol | Cholesterol | C00187 | 329.3 | 47.79 | EI+ | 386.354858 | - | 841 |
| Sterol | β-Sitosterol | C01753 | 207.0 | 49.45 | EI+ | 414.386169 | - | 782 |
| Sugar | α-D-Glucose 6-phosphate | C00668 | 259.095764 | 1.35 | ESI− | 260.029724 | 0.052061 | - |
| Sugar | Xylobiose | C01630 | 281.136261 | 1.21 | ESI− | 282.095093 | 2.008703 | - |
| Sugar | Sedoheptulose 7-phosphate | C05382 | 289.038208 | 1.37 | ESI− | 290.040283 | 0.678267 | - |
| Sugar | *N*-Acetyl-D-glucosamine 6-phosphate | C00357 | 300.021881 | 1.48 | ESI− | 301.056274 | 0.451413 | - |
| Sugar | Acetyl-maltose | C02130 | 383.086243 | 1.38 | ESI− | 384.12677 | 0.591104 | - |
| Sugar | Trehalose 6-phosphate | C00689 | 421.048401 | 1.35 | ESI− | 422.08255 | 1.477948 | - |
| Sugar | D-Arabinose 5-phosphate | C01112 | 231.026337 | 1.42 | ESI+ | 230.01915 | 0.273358 | - |
| Sugar | D-Glucosamine 6-phosphate | C00352 | 260.053040 | 1.37 | ESI+ | 259.045715 | 0.185105 | - |
| Sugar | D-Lyxose | C00476 | 73.0 | 25.90 | EI+ | 150.052826 | - | 737 |
| Sugar | D-Allose | C01487 | 73.1 | 30.94 | EI+ | 180.063385 | - | 785 |
| Sugar | α,α-Trehalose | C01083 | 361.2 | 45.20 | EI+ | 342.116211 | - | 878 |
| Sugar | Turanose | C19636 | 217.1 | 45.99 | EI+ | 342.116211 | - | 764 |
| Sugar | D-Talose | C06467 | 319.1 | 30.94 | EI+ | 180.063385 | - | 799 |
| Vitamin | Thiamine | C00378 | 335.042786 | 1.43 | ESI− | 265.111755 | 2.140097 | - |
| Vitamin | Riboflavin | C00255 | 375.148285 | 8.57 | ESI− | 376.138275 | 2.388937 | - |
| Vitamin | Riboflavin | C00255 | 377.145782 | 8.57 | ESI+ | 376.138275 | 0.613792 | - |

**Table S2.** Pathway analysis related to the identified metabolites.

| Pathway ID | Pathway Name | Numbers of Metabolites | Category |
|---|---|---|---|
| ko01100 | Metabolic pathways | 73 | Other pathway |
| ko01110 | Biosynthesis of secondary metabolites | 40 | Other pathway |
| ko01060 | Biosynthesis of plant secondary metabolites | 26 | Other pathway |
| ko01130 | Biosynthesis of antibiotics | 25 | Other pathway |
| ko02010 | ABC transporters | 23 | Environmental Information Processing; Membrane transport |
| ko01230 | Biosynthesis of amino acids | 20 | Metabolism; Overview |
| ko01120 | Microbial metabolism in diverse environments | 18 | Other pathway |
| ko04974 | Protein digestion and absorption | 16 | Organismal Systems; Digestive system |
| ko05230 | Central carbon metabolism in cancer | 15 | Human Diseases; Cancers |
| ko00230 | Purine metabolism | 15 | Metabolism; Nucleotide metabolism |
| ko00970 | Aminoacyl-tRNA biosynthesis | 13 | Genetic Information Processing; Translation |
| ko04978 | Mineral absorption | 11 | Organismal Systems; Digestive system |
| ko01070 | Biosynthesis of plant hormones | 10 | Other pathway |
| ko01210 | 2-Oxocarboxylic acid metabolism | 9 | Metabolism; Overview |
| ko01065 | Biosynthesis of alkaloids derived from histidine and purine | 9 | Other pathway |
| ko02060 | Phosphotransferase system (PTS) | 8 | Environmental Information Processing; Membrane transport |
| ko00270 | Cysteine and methionine metabolism | 8 | Metabolism; Amino acid metabolism |
| ko01200 | Carbon metabolism | 7 | Metabolism; Overview |
| ko00051 | Fructose and mannose metabolism | 6 | Metabolism; Carbohydrate metabolism |
| ko00908 | Zeatin biosynthesis | 6 | Metabolism; Metabolism of terpenoids and polyketides |
| ko00240 | Pyrimidine metabolism | 6 | Metabolism; Nucleotide metabolism |
| ko01063 | Biosynthesis of alkaloids derived from shikimate pathway | 6 | Other pathway |
| ko04152 | AMPK signaling pathway | 5 | Environmental Information Processing; Signal transduction |
| ko04080 | Neuroactive ligand-receptor interaction | 5 | Environmental Information Processing; Signaling molecules and interaction |

| | | | |
|---|---|---|---|
| ko05012 | Parkinson's disease | 5 | Human Diseases; Neurodegenerative diseases |
| ko00250 | Alanine, aspartate and glutamate metabolism | 5 | Metabolism; Amino acid metabolism |
| ko00966 | Glucosinolate biosynthesis | 5 | Metabolism; Biosynthesis of other secondary metabolites |
| ko00460 | Cyanoamino acid metabolism | 5 | Metabolism; Metabolism of other amino acids |
| ko04976 | Bile secretion | 5 | Organismal Systems; Digestive system |
| ko01061 | Biosynthesis of phenylpropanoids | 5 | Other pathway |
| ko01064 | Biosynthesis of alkaloids derived from ornithine, lysine and nicotinic acid | 5 | Other pathway |
| ko04024 | cAMP signaling pathway | 4 | Environmental Information Processing; Signal transduction |
| ko04068 | FoxO signaling pathway | 4 | Environmental Information Processing; Signal transduction |
| ko00220 | Arginine biosynthesis | 4 | Metabolism; Amino acid metabolism |
| ko00260 | Glycine, serine and threonine metabolism | 4 | Metabolism; Amino acid metabolism |
| ko00290 | Valine, leucine and isoleucine biosynthesis | 4 | Metabolism; Amino acid metabolism |
| ko00310 | Lysine degradation | 4 | Metabolism; Amino acid metabolism |
| ko00330 | Arginine and proline metabolism | 4 | Metabolism; Amino acid metabolism |
| ko00261 | Monobactam biosynthesis | 4 | Metabolism; Biosynthesis of other secondary metabolites |
| ko00052 | Galactose metabolism | 4 | Metabolism; Carbohydrate metabolism |
| ko00630 | Glyoxylate and dicarboxylate metabolism | 4 | Metabolism; Carbohydrate metabolism |
| ko00640 | Propanoate metabolism | 4 | Metabolism; Carbohydrate metabolism |
| ko00592 | alpha-Linolenic acid metabolism | 4 | Metabolism; Lipid metabolism |
| ko00480 | Glutathione metabolism | 4 | Metabolism; Metabolism of other amino acids |
| ko04977 | Vitamin digestion and absorption | 4 | Organismal Systems; Digestive system |
| ko04918 | Thyroid hormone synthesis | 4 | Organismal Systems; Endocrine system |
| ko04922 | Glucagon signaling pathway | 4 | Organismal Systems; Endocrine system |
| ko04924 | Renin secretion | 4 | Organismal Systems; Endocrine system |
| ko01066 | Biosynthesis of alkaloids derived from terpenoid and polyketide | 4 | Other pathway |
| ko04022 | cGMP-PKG signaling pathway | 3 | Environmental Information Processing; Signal transduction |
| ko04122 | Sulfur relay system | 3 | Genetic Information Processing; Folding, sorting and degradation |

| | | | |
|---|---|---|---|
| ko05231 | Choline metabolism in cancer | 3 | Human Diseases; Cancers |
| ko01502 | Vancomycin resistance | 3 | Human Diseases; Drug resistance |
| ko00280 | Valine, leucine and isoleucine degradation | 3 | Metabolism; Amino acid metabolism |
| ko00350 | Tyrosine metabolism | 3 | Metabolism; Amino acid metabolism |
| ko00360 | Phenylalanine metabolism | 3 | Metabolism; Amino acid metabolism |
| ko00010 | Glycolysis/Gluconeogenesis | 3 | Metabolism; Carbohydrate metabolism |
| ko00500 | Starch and sucrose metabolism | 3 | Metabolism; Carbohydrate metabolism |
| ko00520 | Amino sugar and nucleotide sugar metabolism | 3 | Metabolism; Carbohydrate metabolism |
| ko00190 | Oxidative phosphorylation | 3 | Metabolism; Energy metabolism |
| ko00540 | Lipopolysaccharide biosynthesis | 3 | Metabolism; Glycan biosynthesis and metabolism |
| ko00071 | Fatty acid degradation | 3 | Metabolism; Lipid metabolism |
| ko00564 | Glycerophospholipid metabolism | 3 | Metabolism; Lipid metabolism |
| ko01040 | Biosynthesis of unsaturated fatty acids | 3 | Metabolism; Lipid metabolism |
| ko04923 | Regulation of lipolysis in adipocytes | 3 | Organismal Systems; Endocrine system |
| ko04925 | Aldosterone synthesis and secretion | 3 | Organismal Systems; Endocrine system |
| ko04740 | Olfactory transduction | 3 | Organismal Systems; Sensory system |
| ko01062 | Biosynthesis of terpenoids and steroids | 3 | Other pathway |
| ko04142 | Lysosome | 2 | Cellular Processes; Transport and catabolism |
| ko02020 | Two-component system | 2 | Environmental Information Processing; Signal transduction |
| ko04150 | mTOR signaling pathway | 2 | Environmental Information Processing; Signal transduction |
| ko04151 | PI3K-Akt signaling pathway | 2 | Environmental Information Processing; Signal transduction |
| ko05200 | Pathways in cancer | 2 | Human Diseases; Cancers |
| ko05032 | Morphine addiction | 2 | Human Diseases; Substance dependence |
| ko05034 | Alcoholism | 2 | Human Diseases; Substance dependence |
| ko00380 | Tryptophan metabolism | 2 | Metabolism; Amino acid metabolism |
| ko00400 | Phenylalanine, tyrosine and tryptophan biosynthesis | 2 | Metabolism; Amino acid metabolism |
| ko00960 | Tropane, piperidine and pyridine alkaloid biosynthesis | 2 | Metabolism; Biosynthesis of other secondary metabolites |
| ko00020 | Citrate cycle (TCA cycle) | 2 | Metabolism; Carbohydrate metabolism |

| | | | |
|---|---|---|---|
| ko00030 | Pentose phosphate pathway | 2 | Metabolism; Carbohydrate metabolism |
| ko00040 | Pentose and glucuronate interconversions | 2 | Metabolism; Carbohydrate metabolism |
| ko00620 | Pyruvate metabolism | 2 | Metabolism; Carbohydrate metabolism |
| ko00650 | Butanoate metabolism | 2 | Metabolism; Carbohydrate metabolism |
| ko00195 | Photosynthesis | 2 | Metabolism; Energy metabolism |
| ko00680 | Methane metabolism | 2 | Metabolism; Energy metabolism |
| ko00710 | Carbon fixation in photosynthetic organisms | 2 | Metabolism; Energy metabolism |
| ko00720 | Carbon fixation pathways in prokaryotes | 2 | Metabolism; Energy metabolism |
| ko00920 | Sulfur metabolism | 2 | Metabolism; Energy metabolism |
| ko00100 | Steroid biosynthesis | 2 | Metabolism; Lipid metabolism |
| ko00120 | Primary bile acid biosynthesis | 2 | Metabolism; Lipid metabolism |
| ko00140 | Steroid hormone biosynthesis | 2 | Metabolism; Lipid metabolism |
| ko00561 | Glycerolipid metabolism | 2 | Metabolism; Lipid metabolism |
| ko00730 | Thiamine metabolism | 2 | Metabolism; Metabolism of cofactors and vitamins |
| ko00740 | Riboflavin metabolism | 2 | Metabolism; Metabolism of cofactors and vitamins |
| ko00770 | Pantothenate and CoA biosynthesis | 2 | Metabolism; Metabolism of cofactors and vitamins |
| ko00790 | Folate biosynthesis | 2 | Metabolism; Metabolism of cofactors and vitamins |
| ko00860 | Porphyrin and chlorophyll metabolism | 2 | Metabolism; Metabolism of cofactors and vitamins |
| ko00410 | β-Alanine metabolism | 2 | Metabolism; Metabolism of other amino acids |
| ko01212 | Fatty acid metabolism | 2 | Metabolism; Overview |
| ko01220 | Degradation of aromatic compounds | 2 | Metabolism; Overview |
| ko00984 | Steroid degradation | 2 | Metabolism; Xenobiotics biodegradation and metabolism |
| ko04973 | Carbohydrate digestion and absorption | 2 | Organismal Systems; Digestive system |
| ko04913 | Ovarian steroidogenesis | 2 | Organismal Systems; Endocrine system |
| ko04917 | Prolactin signaling pathway | 2 | Organismal Systems; Endocrine system |
| ko04611 | Platelet activation | 2 | Organismal Systems; Immune system |
| ko04723 | Retrograde endocannabinoid signaling | 2 | Organismal Systems; Nervous system |
| ko04066 | HIF-1 signaling pathway | 1 | Environmental Information Processing; Signal transduction |

| | | | |
|---|---|---|---|
| ko04070 | Phosphatidylinositol signaling system | 1 | Environmental Information Processing; Signal transduction |
| ko04071 | Sphingolipid signaling pathway | 1 | Environmental Information Processing; Signal transduction |
| ko04340 | Hedgehog signaling pathway | 1 | Environmental Information Processing; Signal transduction |
| ko05215 | Prostate cancer | 1 | Human Diseases; Cancers |
| ko05217 | Basal cell carcinoma | 1 | Human Diseases; Cancers |
| ko04931 | Insulin resistance | 1 | Human Diseases; Endocrine and metabolic diseases |
| ko05320 | Autoimmune thyroid disease | 1 | Human Diseases; Immune diseases |
| ko05120 | Epithelial cell signaling in Helicobacter pylori infection | 1 | Human Diseases; Infectious diseases |
| ko05132 | Salmonella infection | 1 | Human Diseases; Infectious diseases |
| ko05142 | Chagas disease (American trypanosomiasis) | 1 | Human Diseases; Infectious diseases |
| ko05143 | African trypanosomiasis | 1 | Human Diseases; Infectious diseases |
| ko05146 | Amoebiasis | 1 | Human Diseases; Infectious diseases |
| ko05014 | Amyotrophic lateral sclerosis (ALS) | 1 | Human Diseases; Neurodegenerative diseases |
| ko05030 | Cocaine addiction | 1 | Human Diseases; Substance dependence |
| ko05031 | Amphetamine addiction | 1 | Human Diseases; Substance dependence |
| ko00340 | Histidine metabolism | 1 | Metabolism; Amino acid metabolism |
| ko00311 | Penicillin and cephalosporin biosynthesis | 1 | Metabolism; Biosynthesis of other secondary metabolites |
| ko00331 | Clavulanic acid biosynthesis | 1 | Metabolism; Biosynthesis of other secondary metabolites |
| ko00332 | Carbapenem biosynthesis | 1 | Metabolism; Biosynthesis of other secondary metabolites |
| ko00401 | Novobiocin biosynthesis | 1 | Metabolism; Biosynthesis of other secondary metabolites |
| ko00521 | Streptomycin biosynthesis | 1 | Metabolism; Biosynthesis of other secondary metabolites |
| ko00901 | Indole alkaloid biosynthesis | 1 | Metabolism; Biosynthesis of other secondary metabolites |
| ko00950 | Isoquinoline alkaloid biosynthesis | 1 | Metabolism; Biosynthesis of other secondary metabolites |
| ko00965 | Betalain biosynthesis | 1 | Metabolism; Biosynthesis of other secondary metabolites |
| ko00053 | Ascorbate and aldarate metabolism | 1 | Metabolism; Carbohydrate metabolism |
| ko00562 | Inositol phosphate metabolism | 1 | Metabolism; Carbohydrate metabolism |
| ko00550 | Peptidoglycan biosynthesis | 1 | Metabolism; Glycan biosynthesis and metabolism |
| ko00061 | Fatty acid biosynthesis | 1 | Metabolism; Lipid metabolism |

| | | | |
|---|---|---|---|
| ko00062 | Fatty acid elongation | 1 | Metabolism; Lipid metabolism |
| ko00073 | Cutin, suberine and wax biosynthesis | 1 | Metabolism; Lipid metabolism |
| ko00565 | Ether lipid metabolism | 1 | Metabolism; Lipid metabolism |
| ko00600 | Sphingolipid metabolism | 1 | Metabolism; Lipid metabolism |
| ko00130 | Ubiquinone and other terpenoid-quinone biosynthesis | 1 | Metabolism; Metabolism of cofactors and vitamins |
| ko00760 | Nicotinate and nicotinamide metabolism | 1 | Metabolism; Metabolism of cofactors and vitamins |
| ko00780 | Biotin metabolism | 1 | Metabolism; Metabolism of cofactors and vitamins |
| ko00830 | Retinol metabolism | 1 | Metabolism; Metabolism of cofactors and vitamins |
| ko00430 | Taurine and hypotaurine metabolism | 1 | Metabolism; Metabolism of other amino acids |
| ko00450 | Selenocompound metabolism | 1 | Metabolism; Metabolism of other amino acids |
| ko00472 | D-Arginine and D-Ornithine metabolism | 1 | Metabolism; Metabolism of other amino acids |
| ko00473 | D-Alanine metabolism | 1 | Metabolism; Metabolism of other amino acids |
| ko00909 | Sesquiterpenoid and triterpenoid biosynthesis | 1 | Metabolism; Metabolism of terpenoids and polyketides |
| ko00981 | Insect hormone biosynthesis | 1 | Metabolism; Metabolism of terpenoids and polyketides |
| ko01057 | Biosynthesis of type II polyketide products | 1 | Metabolism; Metabolism of terpenoids and polyketides |
| ko00361 | Chlorocyclohexane and chlorobenzene degradation | 1 | Metabolism; Xenobiotics biodegradation and metabolism |
| ko00624 | Polycyclic aromatic hydrocarbon degradation | 1 | Metabolism; Xenobiotics biodegradation and metabolism |
| ko00642 | Ethylbenzene degradation | 1 | Metabolism; Xenobiotics biodegradation and metabolism |
| ko00643 | Styrene degradation | 1 | Metabolism; Xenobiotics biodegradation and metabolism |
| ko00791 | Atrazine degradation | 1 | Metabolism; Xenobiotics biodegradation and metabolism |
| ko04975 | Fat digestion and absorption | 1 | Organismal Systems; Digestive system |
| ko04919 | Thyroid hormone signaling pathway | 1 | Organismal Systems; Endocrine system |
| ko04721 | Synaptic vesicle cycle | 1 | Organismal Systems; Nervous system |
| ko04726 | Serotonergic synapse | 1 | Organismal Systems; Nervous system |
| ko04727 | GABAergic synapse | 1 | Organismal Systems; Nervous system |
| ko04728 | Dopaminergic synapse | 1 | Organismal Systems; Nervous system |
| ko04742 | Taste transduction | 1 | Organismal Systems; Sensory system |
| ko04744 | Phototransduction | 1 | Organismal Systems; Sensory system |

| | | | |
|---|---|---|---|
| ko04750 | Inflammatory mediator regulation of TRP channels | 1 | Organismal Systems; Sensory system |
